# The context, need, limitations, and delivery of children and young people's social prescribing

**DOI:** 10.1111/dmcn.16130

**Published:** 2024-10-16

**Authors:** Kerryn Husk, Vashti Berry

**Affiliations:** ^1^ University of Plymouth Faculty of Health Plymouth UK; ^2^ University of Exeter Children and Young People's Mental Health Research Collaboration (ChYMe) Exeter UK

## Abstract

This commentary is on the original article by Ostojic et al. on pages 223–234 of this issue.

In principle, social prescribing offers the potential to mitigate the impact of rising presentations of young people with mental health issues on already stretched statutory services. However, we know that the universal all‐age offer of social prescribing is often not taken up by those most in need, including children and young people. Thus, an offer targeted and tailored towards children and young people is appropriate^1^ (as it has been with, for example, older adults) but with variation in interpretation and implementation, including entry points, link worker placement, and community‐based activities.

The existing evidence base for interventions to support the mental health of children with multiple and long‐term conditions is limited.^2^ Research focused on children and young people's experiences of such programmes suggests a need to design and test interventions that centre on building hope and empowerment, by allowing individuals to share their experiences and to build relationships and social support.^3^ The study by Ostojic et al. offers this potential.^4^

Social prescribing is no panacea and there is the potential to perpetuate or exacerbate existing underlying issues with health service delivery. First, there is often a concern that social prescribing offers replace or delay access to more appropriate services such as acute mental health care. Second, the breadth of eligibility (even with condition‐specific cohorts) leads to complication around access with the potential to widen existing inequalities as offers are not targeted. Third, it is important to reflect on the funding structures of the voluntary, community, and social enterprise sector offering activities through social prescribing pathways; these are often short‐term and funded by grants, with related employment precarity.^5^ In addition, community assets for specific health conditions may be specialist voluntary, community, and social enterprise rather than general groups, and more likely to be concentrated in urban rather than rural and coastal areas. Fourth, it is not common to see such programmes placing young people at the centre of design, with delivery often policy‐led rather than needs‐led, so the current study is a positive step. Fifth, there is a difficult line to make between unmet social needs and mental health difficulties. Linked to the first point, social prescribing is not appropriate for significant mental health needs; it is a promotion, prevention, or early intervention approach and not designed to address clinical mental health disorders that might be comorbid with physical health conditions. Lastly, the study by Ostojic et al. suggests that the next step should be a randomized controlled trial. There is no mention of cost, but clearly economic considerations will be important when adding pathways to secondary care. More generally, the evidence needs in this area are complex, and defining the range of relevant outcomes to be measured will be critical.

We know from existing research into the link worker role that facilitating diverse entry points to social prescribing is supported by the link workers themselves and in other sectors. The question remains as to whether it is most appropriate to engage hospital‐based link workers to enable these social prescribing pathways, or whether existing community‐based link workers should extend their already diverse role. Additionally, despite the complexities described above there is the potential for a more robust study design in a contained population which would address some of the fundamental criticisms of the evidence relating to social prescribing. Finally, the study by Ostojic et al. considers whether the family model of social prescribing may work best with younger children with health conditions, owing to the high likelihood of parental needs.

## Data Availability

Not required.
